# The Prevalence and Correlates of Social Anxiety Symptoms among People with Schizophrenia in Ethiopia: An Institution-Based Cross-Sectional Study

**DOI:** 10.1155/2020/3934680

**Published:** 2020-03-24

**Authors:** Boki Kibru, Getachew Tesfaw, Demeke Demilew, Endalamaw Salelew

**Affiliations:** ^1^Gefersa Mental Health Rehabilitation Centre, Addis Ababa, Ethiopia; ^2^Department of Psychiatry, College of Medicine and Health Science, University of Gondar, Gondar, Ethiopia

## Abstract

**Background:**

The comorbidity of social anxiety disorder is very common in schizophrenia patients and affects almost all age groups. This social anxiety disorder negatively impacts the quality of life, medication adherence, and treatment outcomes of people with schizophrenia. It is not well recognized in clinical settings. Therefore, assessing social anxiety symptoms and its associated factors was significant to early intervention and management of schizophrenia patients in Ethiopia.

**Methods:**

An institution-based cross-sectional study was conducted at Amanuel Mental Specialized Hospital in Addis Ababa, Ethiopia. Data collectors randomly recruited 423 schizophrenic patients by using the systematic sampling technique. A face-to-face interviewer-administered questionnaire was used to collect data. The standardized Liebowitz Social Anxiety Scale (LSAS) was employed to assess individual social anxiety symptoms. We computed bivariate and multivariate binary logistic regressions to identify factors associated with social anxiety symptoms. Statistical significance was declared at *p* < 0.05.

**Results:**

The prevalence of social anxiety symptoms was 36.2% (95% CI: 31.50, 40.80). Male sex (AOR = 2.03, 95% CI: 1.20, 3.44), age of onset of schizophrenia (AOR = 1.91, 95% CI:1.17, 3.12), positive symptoms (AOR = 0.75, 95% CI:0.67, 0.83), depression/anxiety symptoms (AOR = 1.29, 95% CI: 1.18, 1.41), number of hospitalizations (AOR = 2.80, 95% CI:1.32, 5.80), and suicidal ideation (AOR = 0.44, 95% CI: 0.26, 0.74) were factors significantly associated with social anxiety symptoms at *p* < 0.05. *p* < 0.05.

**Conclusion:**

The prevalence of social anxiety symptoms among schizophrenia patients was found to be high. Timely treatment of positive and depression/anxiety symptoms and suicide risk assessments and interventions need to be done to manage the problems.

## 1. Introduction

Schizophrenia is a severe and disabling chronic mental disorder characterized by deficits in the thought process, perception, and emotional responsiveness [1]. Between 13.1 and 20.9 million people were estimated to have schizophrenia globally from 1990-2016 [2], and it will be the seventh leading cause of disability by 2020 [3], with its lifetime prevalence estimated at approximately 1% [1]. In Ethiopia, 90% of people living with schizophrenia did not get modern mental health services [4]. A cohort study conducted among 321 schizophrenia patients on clinical outcome for a mean duration of 3.4 years follow-ups; one-third of participants were persistently ill and the remaining patients had an episodic course [4]. Another community-based cross-sectional study done on the attitude about schizophrenia revealed that 62.7% had a negative attitude towards schizophrenia [5].

Social anxiety disorder (SAD) whose lifetime prevalence ranges from 3 to 13% is another intense and persistent problem that causes embarrassment to sufferers in social situations [1, 2]. Cultural differences have a strong contribution on anxiety disorders in different countries [6]. It profoundly interferes with the life of a person in many areas by affecting academic achievement, occupational competence, and social contact [1]. In Ethiopia, the prevalence of social anxiety disorder was found to be 63% among persons aged 12-17 years and it was significantly associated with female gender, low educational status, sexual victimization, and experience of peer victimization [7]. Another two different studies conducted on social anxiety disorder among university students in Ethiopia revealed a 31.2% [8] and 32.8% [9] prevalence. The comorbidity of SAD with schizophrenia is common, and it is the most frequent type of anxiety disorder in schizophrenia patients [10–15]. The rates of social anxiety symptoms among schizophrenia patients were reported to be within the range of 4.76%-67% [15, 16].

Even though social anxiety symptoms are very common in schizophrenia patients, it is not well recognized in clinical settings [17]. In particular, in the first episode of schizophrenia, there is a high comorbidity with social anxiety disorder [14]. Negative symptoms and low self-esteem are also consistently correlated with social anxiety symptoms among schizophrenia patients [18, 19]. These disorders are more frequently observed in patients who have a history of childhood sexual abuse, separation anxiety, and school phobia [15, 20]. Comorbid SAD with schizophrenia negatively affects treatment outcomes and prognosis of the disorder [20]. These comorbid patients have a greater lethality to suicide attempts, lower quality of life, history of substance abuse, and poor social adjustment [17].

Several factors appear to influence the risk of social anxiety among individuals with schizophrenia. These include duration of illness, multiple episodes, low quality of life, impaired function, relapse, suicide attempts, number of hospitalizations, severity of the symptoms, and disability [20–24]. Evidence suggests that social anxiety contributes to a serious decrease in treatment outcomes and functional impairments [11, 25].

Although the comorbidity of social anxiety symptoms with schizophrenia is associated with barriers to respectively treatment including increased risk of suicide, treatment resistance, greater chance of for recurrence, and relapse among schizophrenia patients, to the best of our knowledge, there has been no published study on social anxiety symptoms and associated factors among schizophrenia patients in Ethiopia. Therefore, this study will close the gap by identifying solutions and assessing the prevalence and factors associated with social anxiety symptoms among people with schizophrenia.

### 1.1. Objective

The aim of this study was to assess the prevalence of social anxiety symptoms and associated factors among people with schizophrenia at Amanuel Mental Specialized hospital, Addis Ababa, Ethiopia, 2018.

## 2. Methods and Materials

### 2.1. Study Setting and Period

An institution-based cross-sectional study was conducted from May–June 2018 at Amanuel Mental Specialized Hospital, Addis Ababa, Ethiopia. It is the only mental hospital in the country with 300 beds and 3420 schizophrenia patients on monthly follow-ups.

### 2.2. Study Population

All schizophrenia outpatients aged ≥18 were included, while patients found to be severely ill (incoherent and actively psychotic) were excluded.

### 2.3. Sampling Procedures

The sample size was calculated by using the single population proportion formula with a 95% CI, a 5% margin of error, and social anxiety symptoms of 50% due to lack of published work in Ethiopia. Having assumed a 10% nonresponse rate, 423 schizophrenic patients were recruited randomly by using the systematic sampling technique. The sampling interval was determined by dividing the total study population who had follow-ups during the month of the data collection period at the psychiatry OPD at AMSH by the total sample size. The selection skips interval was eight; so, the participants were selected at every eighth interval. The first individual was selected by the lottery method from the appointment register. If the selected individual was not present or refused to participate, the data collector will take the next participant and continue the interval from the interviewed participant.

### 2.4. Data Collection

Data were collected using a pretested interviewer-administered questionnaire which contained social anxiety as the dependent variable and several other explanatory variables, including sociodemographic factors, social support, clinical factors, and substance use.

### 2.5. Measurements

Social support was collected by the Oslo 3-item social support scale, which has a 3-item questionnaire commonly used to assess social support in several previous studies. The sum score scale ranged from 3 to 14 and had three broad categories: “poor support” 3-8, “moderate support” 9-11, and “strong support” 12-14 [26]. It has been used in Ethiopia in different clinical settings [27–29].

Suicidal ideation and attempts were measured according to the WHO questionnaire. If the respondent answered “Yes” to the question, “Have you ever seriously thought about committing suicide or have attempted suicide?” they were considered to have suicidal ideation made attempts [30]. The PANSS symptom severity groups were assessed using a five-factor model which breaks down the symptoms into positive, negative, cognitive/disorganization, depression/anxiety, and excitement/hostility components which explained the scale structure better than the original solution. The PANSS five-factor structure represented a more valid distribution of the items than the original three-factor solution, and agreements across studies could be reached for many items which represented each of the five factors [31]. The tool was tested on schizophrenia patients in a previous study in Ethiopia [32]. Social anxiety symptoms were measured using the Liebowitz Social Anxiety Scale Self-Reporting (LSAS-SR) questionnaire with cut-off points of 30 as it provided the best balance of sensitivity and specificity. It had 24 items divided into subscales. The first subscale which measured difficulty with social interaction had 11 items, while the second subscale which measured difficulty with performance had 13 items. The self-reporting LSAS total score reliability was 0.83 [33, 34].

### 2.6. Data Processing and Analysis

Data were entered into EpiData software version 3.1 and imported to SPSS version 21 for analysis. Univariate, bivariate, and multivariate logistic regression analyses were done to see the association of each independent variable with the outcome variable. The strength of associations was evaluated using the adjusted odds ratio with a 95% CI, and a *p* value less than 0.05 was considered statistically significant.

#### 2.6.1. Ethical Considerations

Ethical approval was obtained from the Institutional Review Board (IRB) of the University of Gondar. Letter of permission was issued by Amanuel Mental Specialized Hospital. We received informed written consent from the study participants. Confidentiality was maintained by omitting personal identifiers.

## 3. Results

### 3.1. Sociodemographic Characteristics

Out of a total of 423 participants, 409 completed the survey with a response rate of 96.7%. The mean age of the respondents was 22 (±9.73) years, and nearly two-fifths (37.6%) were between the ages of 18 and 25 years. Of the participants, 255 (62.3%) were male and 242 (59.2%) were single. Almost half (50.9%) of the participants were Orthodox Christian; two-fifths (40.4%) were Oromo, and 170 (41.6%) had a primary level education (Table 1).

### 3.2. Clinical Characteristics of Study Participants

Of the 423 participants, 53% (*n* = 215) had age of onsets of schizophrenia after 25 years. More than half (56%) of the respondents had 6-10 years duration of illness, and nearly one-third (28.1%) had less than and equal to two admissions. Almost half of the participants (49.4%) had suicidal ideation, and over half (51.3%) suicide attempts. In this result, the overall PANSS score mean and standard deviation was 43.68 (±8.16). Specifically, the mean and standard deviation of positive, negative, and depression/anxiety symptoms were 8.57 ± 2.69, 9.24 ± 2.64, and 7.7 ± 3.00, respectively, (Table 2).

### 3.3. Social and Substance-Related Factors

Regarding social factors, 191 (46.7%) and 186 (45.5%) of the participants had moderate and poor social support, respectively. At the moment, over two-thirds (63.8%) were smoking tobacco, 57.7% (*n* = 236) were drinking alcohol, and about two-thirds (59.2%) were chewing chat and almost all of the participants were lifetime users of the substances (Table 3).

### 3.4. The Prevalence of Social Anxiety Symptoms

This study showed that the prevalence of social anxiety symptoms among participants was 36.2%, with a 95% CI (31.50, 40.80). Of the participants, one-fifth (18.66%), one-tenth (11.78%), and 5.77% had mild, moderate, and severe social anxiety symptoms, respectively (Table 4).

### 3.5. Factors Associated with Social Anxiety Symptoms

Out of the independent variables, male sex, age of onset of schizophrenia, presence of positive symptoms, negative symptoms, depression/anxiety symptoms, disorganized behavior, number of hospitalizations, poor social support, suicidal ideation, and attempts yielded *p* values below 0.2 in the bivariate logistic regression and were considered for the multivariate logistic regression model. The multivariate analysis suggested that age of onset of schizophrenia before 25 years had a 1.93 times (95% CI: 1.18, 3.19) greater likelihood of developing social anxiety symptoms compared to their counterparts. Male sex was two times (95% CI: 1.20, 3.44) more likely to have anxiety symptoms compared to female sex. Participants who were hospitalized more than two times had a 2.8-fold (95% CI: 1.32, 5.80) increased risk for social anxiety symptoms compared to those who had no history of admission. The positive symptoms of schizophrenia increased by one unit, while every social anxiety symptom decreased by 0.75 units, and depression/anxiety symptoms of schizophrenia increased by one unit, while every social anxiety symptom increased by 1.28 units. People reporting poor social support had a 5.47-fold increased (95% CI: 2.03, 14.70) risk for social anxiety symptoms compared to patients with good social support; patients with suicidal ideation were also associated with social anxiety symptoms. The odds of social anxiety symptoms increased by two times (95% CI: 1.26, 3.26) for patients who had suicidal ideation compared to patients who did not have the problem. Finally, the risk for social anxiety symptoms for patients who made suicide attempts increased by 1.93 times (95% CI: 1.14, 3.26) compared with patients who did not make such attempts (Table 5).

## 4. Discussion

In this study, the prevalence of social anxiety symptoms and its possible association with various factors were assessed. The results revealed that a remarkable proportion of social anxiety symptoms were found in people with schizophrenia. The prevalence of social anxiety symptoms among individuals with schizophrenia was found to be 36.2%. This result is consistent with those of other studies carried out in Israel 38% [13], Czech Republic 31.1% [35], Italy 36.3% [17], Canada 32% [36], and India 31.03% [37].

On the other hand, our finding is higher than those of studies done in Nigeria 17% [21], Israel 11% [11], Turkey 4.76% [15], Britain 25% [14], Brazil 17% [25], and Canada 14.9% [38]. The variations in the above rates might be due to differences in the types of study designs, sample sizes, the uses of various scales and ratings for assessing the level of social anxiety symptoms, methodologies, and sociocultural contrasts between Ethiopia and other countries. The prevalence of social anxiety depends on the particular culture and in DSM-V; different cultures have a specific expression of social anxiety known to exist [1, 39]. The prevalence social anxiety is related to different cultural norms across countries [40]. In Ethiopia, traditional beliefs and cultural values should be seen as contributing valuable information about the perceptions and realities of anxiety disorders [41]. So, culture affects the way we express our thoughts, behaviors, and emotions.

Our result is lower than those of studies done in two areas of Canada [16, 42]. The discrepancy might be due to sample size differences, variation in study setting scales for assessing symptoms, and sociocultural distinctions. The study in Canada in the first episode of psychosis among sixty schizophrenia patients assessed through social phobia and anxiety inventory scale questionnaire was 48.8% [42], while in another area of Canada, thirty-six discharged elderly patients with remitted schizophrenia assessed by a five-year longitudinal study using the same tool was high [16].

Male patients were two times at greater risk for social anxiety symptoms than females. In the general population, females were affected more often than males, but in clinical samples, the reverse was often true [1]. In adulthood, the prevalence of anxiety is much higher in females, while schizophrenia shows no consistent sex difference in prevalence although men typically experienced earlier onsets of [43]. A study done in the USA on social anxiety symptoms revealed that there was no gender difference in the prevalence of the anxiety symptoms among schizophrenia patients [44].

Those in age of onset of schizophrenia before 25 years had approximately two times greater likelihood of developing social anxiety symptoms compared to their counterparts. This means that patients with early onsets of schizophrenia feel that they have social anxiety more often than patients with late onsets. The result was in line with that of a study done in the Czech Republic and reported that earlier onsets of schizophrenia were indicators of high vulnerability to social anxiety symptoms [37]. Early onsets of schizophrenia may impair the development of personality, and patients' social roles before they learn how to manage these situations.

Positive symptoms increased by one unit; social anxiety symptoms decreased by 0.75 units. A study conducted in the USA recorded no difference between social anxiety symptoms and positive symptoms for schizophrenia [17], and PANSS positive symptoms subscale was significantly associated with social anxiety symptoms [45]. Our result was different from those of previous studies and needs further research. Depression/anxiety symptoms of schizophrenia increased by one unit, while every social anxiety symptom increased by 1.28 units. The severity of depression/anxiety symptoms among schizophrenia patients led to social anxiety symptoms [45]. The severity of psychotic episode in acute phase of schizophrenia predicted the severity of concurrent depression/anxiety symptoms [46]. The severity of schizophrenia symptoms increased the symptoms of social phobia symptoms [22], and patients diagnosed with comorbid schizophrenia and social anxiety symptoms had significantly higher scores on the mean PANSS [21]. Poor social support was five times a greater risk for social anxiety symptoms than good social support. Comorbidity of social anxiety symptoms worsened social interaction and job performance of schizophrenia patients [17].

Those with suicidal ideation had two times greater likelihood of developing social anxiety symptoms than their counterparts. This was consistent with the finding of a study done in the USA which found that an increased incidence of suicidal ideation and schizophrenia with social anxiety disorders had a high rate of lifetime suicidal ideation and greater lethality of suicide attempts [13, 20].

The risk of social anxiety symptoms for patients with suicide attempts increased by nearly two times compared with patients with no attempts. Suicide attempts have been associated with depression, mood, anxiety, low self-esteem, negative perceptions, and others, like daily alcohol consumption and distress caused by positive symptoms among schizophrenia patients [47]. A possible explanation for this might be the nature of comorbidity with depression, substance use, the severity of symptoms, and perceived stigma towards the illness.

### 4.1. Strengths and Limitations of the Study

As Amanuel is the only mental specialized hospital in Ethiopia, we easily obtained an adequate sample. We found that the cross-sectional design has prevented us from reporting the causal relationships of the associations. This finding is likely only to hint at the complex interactions between social anxiety and explanatory variables (risk factors). Another important limitation of this study is the fact that the LSAS scale was not validated in Ethiopia, although it is widely used as a screening tool for social anxiety in other countries. Another important limitation is that since the patients were schizophrenic, we faced considerable recall bias and the comorbidity of substance abuse was not assessed. Another limitation of this study was that the symptoms of schizophrenia assessed by using the PANSS mean score was very low; therefore, further research about the PANSS symptoms of schizophrenia and associated factors of social anxiety symptoms should be conducted to strengthen and broaden our result.

## 5. Conclusions

In this study, the overall magnitude of social anxiety disorder was 36.2%. Male sex, age of onset of schizophrenia, positive symptoms, depression/anxiety symptoms, poor social support, hospitalization, and suicidal ideation and attempts were factors significantly associated with social anxiety symptoms. A timely treatment of positive and depression/anxiety symptoms are helpful to reduce the problems. Furthermore, suicide risk assessments and interventions had better be done.

## Figures and Tables

**Table 1:** Sociodemographic characteristics of people with schizophrenia attending as outpatients at Amanuel Mental Specialized Hospital, Addis Ababa, Ethiopia, 2018 (*N* = 409).

Variables	Categories	Frequency	Percent
Age	18-25	154	37.6
	26-35	132	32.3
	36-45	81	19.8
	≥46	42	10.3

Sex	Male	255	62.3
	Female	154	37.7

Religion	Orthodox	208	50.9
	Muslim	141	34.5
	Protestant	60	14.6

Marital status	Single	242	59.2
	Married	167	40.8

Ethnicity	Oromo	165	40.4
	Amhara	137	33.6
	Gurage	82	20
	Tigre	25	6

Educational status	Unable to read and write	41	10
	Primary education	170	41.6
	Secondary education	139	34
	Diploma and above	59	14.4

**Table 2:** Description of clinical variables of people with schizophrenia attending as outpatients at AMSH, 2018 (*N* = 409).

Variables	Categories	Frequency	Percent
Age at onset of illness	Before 25 years	192	47
	After 25 years	215	53
Duration of illness	Less than 1 year	75	18
	1-5years	117	29
	6-10 years	127	56
	>10 years	90	23
Number of admissions	No	238	58.2
	≤2	115	28.1
	>2	56	13.7
Suicidal ideation	Yes	202	49.4
	No	207	50.6
Suicide attempts	Yes	210	51.3
	No	199	48.7
PANSS five factors			
Positive symptoms	Mean and SD	8.57 ± 2.69	—
Negative symptoms	Mean and SD	9.24 ± 2.64	—
Depression/anxiety	Mean and SD	7.7 ± 3.00	—
Disorganized	Mean and SD	6.86 ± 2.00	—
Hostility	Mean and SD	5.25 ± 1.37	—

**Table 3:** Lifetime and current substance use among people with schizophrenia attending as outpatients at AMSH, 2018 (*N* = 409).

Variables	Categories	Frequency	Percent
Social support	Poor	186	45.5
	Medium	191	46.7
	Good	32	7.8
Current use			
Tobacco	Yes	261	63.8
	No	148	36.2
Alcohol	Yes	236	57.7
	No	173	42.3
Khat (chat)	Yes	244	59.7
	No	165	40.3
Lifetime use			
Tobacco	Yes	325	79.5
	No	84	20.5
Alcohol	Yes	321	78.5
	No	88	21.5
Khat (chat)	Yes	334	81.7
	No	75	18.3

**Table 4:** The distribution of participants by their response to LSAS-SR among people with schizophrenia at AMSH, Addis Ababa, Ethiopia, 2018 (*n* = 409).

The 24 items of the LSAS-SR scale	None	Mild	Moderate	Severe
	Frequency (%)	Frequency (%)	Frequency (%)	Frequency (%)
Telephoning in public (p)	267 (65.28)	81 (19.8)	47 (11.49)	14 (3.43)
Participating in small group (p)	221 (54)	70 (17.1)	81 (19.8)	39 (9.1)
Eating in public places (p)	300 (73.4)	66 (16.1)	32 (7.8)	11 (2.7)
Drinking with others in public places (p)	290 (70.9)	54 (13.3)	44 (10.7)	21 (5.1)
Talking to people in authority (s)	187 (45.7)	85 (20.8)	79 (19.3)	58 (14.2)
Acting, performing or giving a talk in front of audiences (s)	263 (64.3)	104 (25.4)	27 (6.6)	15 (3.7)
Going to a party (s)	275 (67.2)	70 (17.1)	49 (12)	15 (3.7)
Working while being observed (p)	240 (58.7)	93 (22.7)	46 (11.3)	30 (7.3)
Writing while being observed (p)	280 (68.4)	58 (14.1)	54 (13.3)	17 (4.1)
Calling someone you do not know very well (s)	222 (54.28)	88 (21.51)	64 (15.65)	35 (5.56)
Talking with people you do not know very well (s)	250 (61.2)	82 (20)	48 (11.7)	29 (7.1)
Meeting with strangers (s)	222 (54.3)	86 (21.0)	74 (18.1)	27(6.6)
Urinating in public bathroom (p)	265 (64.8)	70 (17.1)	49 (12)	25 (6.1)
Entering a room when others are already seated (p)	131 (32)	89 (21.8)	115 (28.1)	74 (18.1)
Being a center of attention (s)	279 (68.2)	109 (26.7)	15(3.7)	6 (1.5)
Speaking up at a meeting (p)	137 (33.5)	95 (23.2)	117 (28.6)	60 (14.7)
Taking a test (p)	349 (85.3)	37 (9.0)	14(3.4)	9 (2.2)
Expressing a disagreement or disapproval to people you do not know very well (s)	286 (69.9)	88 (21.5)	27 (6.6)	8 (2)
Looking at a people you do not know very well in the eyes (s)	264 (64.5)	96 (23.5)	40 (9.8)	9 (2.2)
Giving a report to a group (p)	265 (64.8)	83 (20.3)	48 (11.7)	13 (3.2)
Trying to pick up someone (p)	312 (76.3)	56 (13.7)	29 (7.1)	12 (2.9
Returning goods to the store (s)	358 (87.5)	29 (7.1)	10 (2.4)	12 (2.9)
Giving a party (s)	263(64.3)	89 (21.8)	40 (9.8)	17 (4.2)
Resisting a high pressure salesperson (s)	336 (82.2)	54(13.2)	9 (2.2)	10 (2.4)
Total score	6262 (63.79)	1832 (18.66)	1156 (11.78)	566 (5.77)

**Table 5:** Factors associated with social anxiety disorder among people with schizophrenia attending as outpatients at AMSH, 2018 (*N* = 409).

Variables	Categories	Social anxiety symptoms		COR (95% CI)	AOR (95% CI)
		No	Yes		
Sex	Male	146	109	2.20 (1.41, 3.41)	2.03 (1.20, 3.44)^∗∗∗^
	Female	115	39	1 : 00	1 : 00

Onset of illness	Before 25 years	114	80	1.51 (1.01, 2.28)	1.93 (1.18, 3.19)^∗∗∗^
	After 25 years	147	68	1 : 00	1 : 00

Admission	Never	163	75	1 : 00	1 : 00
	≤2 admission	69	46	1.45 (0.91, 2.30)	1.60 (0.90, 2.84)
	>2 admission	29	27	2.02 (1.12, 3.66)	2.80 (1.32, 5.80)^∗∗∗^

PANSS	Positive	8.57 ± 2.69		0.80 (0.72, 0.88)	0.75 (0.67, 0.84)^∗∗∗^
	Negative	9.24 ± 2.64		1.06 (1.00, 1.14)	1.00 (0.91, 1.12)
	Depression/anxiety	7.7 ± 3.00		1.2 (1.11, 1.29)	1.28 (1.16, 1.40)^∗∗∗^
	Disorganized	6.86 ± 2.00		1.1 (1.00, 1.22)	1.07 (0.93, 1.22)

Social support	Poor	89	97	2.4 (1.08, 5.34)	5.47 (2.03, 14.70)^∗∗^
	Medium	150	41	0.60 (0.26, 1.37)	1.07 (0.39, 2.85)
	Good	22	10	1 : 00	1 : 00

Suicidal ideation	Yes	112	90	2.06 (1.40, 3.11)	2.14 (1.26, 3.62)^∗∗∗^
	No	149	58	1 : 00	1 : 00

Suicidal attempt	Yes	98	85	2.24 (1.49, 3.39)	1.93 (1.14, 3.26)^∗∗^
	No	163	63	1 : 00	1 : 00

Note: ^∗∗^*p* value is significant at < 0.01, ^∗∗∗^*p* value is significant at *p* < 0.001. Hosmer and Lemeshow test *p* value = 0.713.

## Data Availability

The dataset during and/or analyzed during the current study available from the corresponding author on reasonable requests.

## Abbreviations

AMSH: Amanuel Mental Specialized Hospital
ADs: Anxiety disorders
AOR: Adjusted odds ratio
DSM-V: Diagnostic Statistical Manual of Fifth Edition, Text Revision
LSAS: Leibowitz Social Anxiety Scale
LYD: Life years' disability
SAD: Social anxiety disorder
SCS: Social comparison scale
SPSS: Statistical Package for Social Science
OPD: Outpatient department
OR: Odds ratio
UK: United Kingdom
UoG: University of Gondar
USA: United States of America
WHO: World Health Organization.
